# Polydimethylsiloxane in Optics

**DOI:** 10.3390/polym18131589

**Published:** 2026-06-26

**Authors:** Sergio Calixto, Roberto Zitzumbo, Mariana Alfaro-Gomez

**Affiliations:** 1 Centro de Investigaciones en Óptica, Loma del Bosque 115, León 37150, GTO, Mexico; 2 Centro de Innovación Aplicada en Tecnologías Competitivas, Omega 201, Col. Industrial Delta, Leon 37545, GTO, Mexico; rzitzumb@ciatec.mx; 3 Departamento de Matemáticas y Física, Universidad Autónoma de Aguascalientes, Av. Universidad 904, Cd. Universitaria, Aguascalientes 20100, AGS, Mexico; mariana.alfaro@edu.uaa.mx

**Keywords:** optics, polydimethylsiloxane, silicone optical components, microfluidics, lab on a chip, sensors

## Abstract

Optics is the science of light, which supports disciplines like biology, medicine, engineering, materials science, chemistry, physics and more. Optics helps to improve diagnostic speed, portable and user-friendly devices, cost efficiency, and sensitivity. Through time, optical components have been made with hard and non-deformable materials. However, traditional optical elements can no longer meet the needs of the market, and new optical elements are needed, such as materials with higher degrees of freedom. A candidate that has been proposed to replace traditional optical materials is polydimethylsiloxane (PDMS or silicone) because it presents suitable characteristics like biocompatibility, nontoxicity, flexibility, non-biodegradability, high transparency in the UV–visible range, low scattering and absorption, easy fabrication, cost-effective relation and more. Many articles have reported the fabrication of optical components with silicone and the use of these components in optical devices. Unfortunately, there is no review that comprehensively covers the field of optics in relation to the application of silicone. The present work is intended as a descriptive overview to provide a clear and accessible review of the topic, rather than a comparative analysis. Articles describing the use of silicone in the fabrication of optical components during the past 20 years were reviewed.

## 1. Introduction

Through time, materials like plastics, crystals, glasses or metals have been used to fabricate optical elements. These materials are hard and non-deformable. Due to this rigidity, the refractive index, size, radius of curvature, focal distance, grating pitch and other parameters remain fixed throughout their lifetimes. It was in the second half of the 1990s that the group of Prof. G.M. Whitesides [1] at Harvard University and the optics group at the National Physical Laboratory in England, headed by Dr. M.C. Hutley [2], used silicone to make optical elements. M.C. Hutley made copies of micro-optical lenses, while G. M. Whitesides made “Elastomeric Optics” like copies of diffraction gratings, mirrors, microlenses and lenses working in transmission or reflection. Later, in 1998, the Harvard group proposed the prototyping of microfluidic systems with microscopic channels [3]. Silicone components are fabricated by casting, embossing, or injection molding. These methods are cost-effective, and the manufacture is fast. Since then, many optical elements have been made with this material.

Here, we review the chemical and physical characteristics of polydimethylsiloxane, hereafter named “silicone” or PDMS, and describe some of its applications in optical components and devices. This article does not intend to show all the components and devices in which silicone has been used; instead, it tries to show silicone’s versatility and adaptation in the fabrication of optical devices. The main silicone characteristics related to optical applications are good transmittance and low absorption and scattering. We extend the review by briefly describing the characteristics and uses of silicone in other bands of the electromagnetic spectrum beyond the visible. Furthermore, as this review focuses on highlighting the applications of silicone throughout a wide range within the optics field, a detailed comparison with classical materials is not included.

The classification of optical components and devices in this article is not rigorous, as they could belong to two or more disciplines. In the subsection sections, the words “passive behavior” and “Active behavior” have been added. The first one means that the optical component cannot be modified. The second one means that there is a liquid inside the hollow silicone structure. This liquid can be modified, making the optical component “Active”.

## 2. Silicone Chemical and Physical Characteristics

Silicone is a soft, deformable, cost-effective material that has a low melting temperature, high mid-infrared absorption, high purity, moisture resistance, thermal stability, flexibility and nontoxic nature. In addition, silicone exhibits weather, heat, and chemical resistance properties and elasticity with shape memory [4,5]. In addition, silicone perfectly copies the concavity and small features of glass surfaces. It has optical transmission to light from the UV to the near-infrared range (300 nm to 800 nm). The optical properties of silicone have also been measured in other bands of the spectrum, like MIR, FIR or THz.

**Figure 1:**
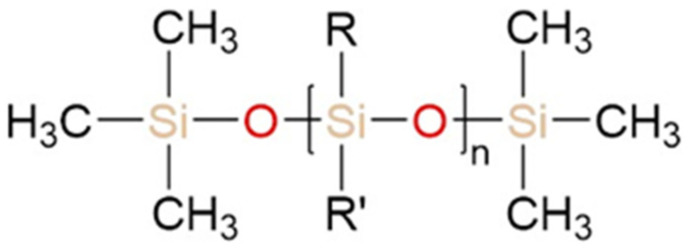
General molecular structure of PDMS. Red O represents oxygen, and yellow Si stands for silicon.

Silicones are inorganic synthetic polymers joined by covalent bonds. In the main chain, silicon atoms bond with oxygen; meanwhile, carbon atoms are bonded at the lateral chains to silicon atoms. The general molecular structure of silicones is represented in Figure 1. Here, the chemical groups R and R’ can be equal or different. When R and R’ are methyl groups (–CH3), the silicone polymer is polydimethylsiloxane (PDMS). 

The molecular structure of silicones has two common chemical groups: the functionality siloxane (–O–Si)n, which is the repeating unit, and the group (Si–O–Si–) that joins the oxygen with the silicon. Both groups are in the main silicon chain. On the one hand, the oxygen–silicon bonds (Si–O–Si) are responsible for the great flexibility in silicone polymers. On the other hand, the amount of repeating units (–O–Si)n determines the polymerization degree to synthesize the silicone prepolymers, with n values between five and 10,000 or more [6].

Silicone prepolymers are low-molecular-weight polymers with poor physical and chemical properties. When a curing agent is added in a 10:1 ratio to silicone prepolymers, a silicone polymer is obtained [7].

The chemical reaction to obtain silicone polymers from prepolymers is the curing reaction, molecular crosslinking or reaction hardening. The curing reaction extension is calculated by the molecular crosslinking density, and it depends on temperature and curing agent concentration [8,9].

The physical and chemical properties of silicone polymers are mainly dependent on the degree of polymerization (n) and on the R and R’ groups, as well as on the molecular crosslinking density.

Based on the above, there is a wide variety of PDMS polymers with similar physical and chemical properties, even though some of the values of the mechanical, thermal and electrical properties vary considerably from one formulation to another.

Cured PDMS polymers exhibit flexible, isotropic, biocompatible properties and are nontoxic and non-biodegradable [10,11]. They are stable and inert in the presence of heat, chemicals and UV radiation [4,12]. PDMS polymers exhibit physical properties that remain relatively stable over a working temperature range of −50 to 150 °C [10]. In addition, they are odorless and colorless [13], hydrophobic, gas-permeable, optically transparent and chemically inert [9,10].

In general, cured PDMS polymers have good chemical, optical, electrical, thermal, and mechanical properties. The hydrophobic character of cured PDMS polymers does not vary significantly with the molecular crosslinking density. However, surface treatment of cured PDMS films, as well as grafting other polymers into the silicone matrix polymer, increases their hydrophilic character [9,14,15]. It is also possible to increase the hydrophobic character of PDMS for lenses and other industrial uses [11].

The properties of cured PDMS can be improved by adding small concentrations of additives to PDMS prepolymers during their synthesis processes or by adding additives to synthesized prepolymers. The most common fillers are organic and inorganic nanometric-sized additives [16,17,18] and nanocarriers that increase some properties such as mechanical, electrical and thermal, among others [10].

In practice, the reinforcing fillers are mixed in a dispersing solution, to which commercial PDMS prepolymers are added before adding the curing agent, and then the molecular crosslinking process is carried out to obtain nanostructures of cured silicone [16].

During the molecular crosslinking process of PDMS prepolymers, covalent bonds are formed, increasing the molecular weight of the prepolymer and transforming it into a cured polymer. That is, the internal molecular structure of the PDMS prepolymer is crosslinked, forming a homogeneous and imperfect crosslinked three-dimensional network because some molecular chains of the silicone prepolymer remain uncrosslinked [18]. In this way, molecular chains with and without molecular crosslinking coexist in the three-dimensional network [19,20,21].

The molecular chains in the three-dimensional network without crosslinking could continue their crosslinking process if the polymer is exposed to a thermal post-curing process, increasing its mechanical properties [9]. Therefore, the values of the physical and chemical properties of the cured silicone polymers that have been reported in the literature (see Table 1) should be considered with caution.

The optical properties of silicone have been studied, and it has been found that the refractive index varies from 1.3 to 1.5 between the UV and the millimeter regions [36]. In particular, PDMS has a refractive index of about 1.31 at 1 THz, presenting dispersion between 0.1 and 1.1 THz. THz absorption was compared to similar polymers [37,38].

## 3. Solid Micro-Optical Silicone Structures

### 3.1. Microlenses and Microlens Arrays (Solid Structures, Passive Behavior)

The word micro-optics describes optical elements with dimensions of a millimeter or smaller, which can include elements such as lenses, mirrors, gratings, polarizers and more. One of the more important elements is the microlens. For microlenses with diameters less than 1 mm, conventional grinding and polishing fabrication methods are impractical. Instead, when materials like plastic and glass are selected, the primary method for microlens fabrication is molding. Another method for microlens fabrication is laser heating using a focusing lens. The laser emission wavelength is strongly absorbed by the material, producing local melting at the focal spot. Then, the microlens appears as a bump in the material. The melting method is generally used to make microlenses from photoresists. This method has also been used with materials like glass [39] and silicone [39,40]. Reference [39] mentions the use of silicone in solid form. After melting, different plano-convex lenses were formed with diameters of about 700 to 1000 microns. Focal distances from about 2 mm to 6 mm were presented. Also, spherical lenses with diameters of about 700 microns can be made with the melting method.

Silicone has also shown versatility in the replication of microstructured optical surfaces, allowing the fabrication of a wide range of microscopic structures like diffusers, micro-prism arrays, Fresnel lenses, microlens arrays and others. Reference [2] mentions the replication of microlens arrays and diffraction gratings with silicone. Microlens arrays were first made by a thermal reflow process using a photoresist with a period of 375 µm and a diameter of 145 µm and then replicated with silicone. Diffraction gratings with a period of 1 µm were also replicated, although at high spatial frequency, some lack of fidelity was noted.

Reference [40] reports the fabrication of divergent microlenses with diameters from about 100 µm to 1 mm using the melting method and a CO_2_ laser (λ = 10.6 µm). The recording material was a thin film of a mixture of silicone and a curing agent, which was placed on a flat glass substrate. After infrared light exposure, the mixture was cured by leaving it at room temperature for ~6 h.

### 3.2. Diffraction Gratings (Solid Structures, Passive Behavior)

A diffraction grating consists of parallel equispaced lines lying in the same plane. This structure diffracts light into various directions called diffracted orders. When light with different wavelengths illuminates the grating, a spectrum is seen.

Diffraction gratings can be fabricated using light interference. If two coherent beams of light intersect at an angle of 2θ, they will generate an interference pattern consisting of sinusoidal fringes with a spacing X within the common volume. The distance between the fringes is the pitch, and it is shorter when the θ angle is large.

**Figure 2:**
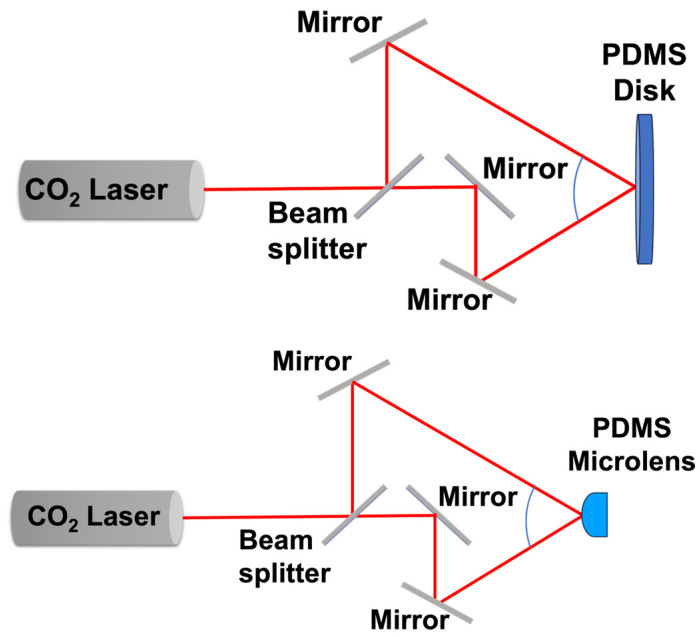
Configurations used to record diffraction gratings over a PDMS disk and over the convex surface of a silicone lens [39,40].

References [39,40] reported an interference method for fabricating diffraction gratings using a CO_2_ laser of infrared light (λ = 10.6 µm) that is absorbed by silicone (Figure 2). Disks of silicone, about 3.5 cm in diameter and about 200 µm thick, were used as substrates to record infrared interference patterns, with exposure times of about 150 ms. Because surface modulation is made by heat, lateral conduction prevents high frequencies from being recorded and low modulations from being achieved. Spatial frequencies of the recorded gratings cannot be more than a few lines per millimeter. Recorded spatial frequencies ranged from 3.5 to 14 lines/mm. The profile of the gratings was measured with a surface analyzer, finding a sinusoidal shape. 

In references [39,40], a hybrid silicone element consisting of a lens and an integrated diffraction grating is presented. The grating was recorded on the convex surface of the lens. When light from a He–Neon laser (λ = 632.8 nm) was sent to the hybrid element, three spots were seen at the focal distance (3.5 mm), meaning light was diffracted by the grating and focused by the lens (Figure 2).

Another diffraction grating fabrication method is photolithographically patterned lines of photoresist on silicon wafers [41,42]. In one instance, after the photoresist patterned lines were fabricated, a silicone film was cast against the pattern, cured and gently peeled away. In this way, transparent samples of the elastomer with binary gratings were embossed on the surface. The grating slits were 2 µm wide, separated by 2 µm.

Recently, a method to make diffraction gratings with a period smaller than the original mold was shown [43]. The method involved the use of PDMS mixed with toluene. The mixture was poured over the surface of a master grating; after solidification, the new PDMS grating structure was demolded, and a uniformly periodic shrinkage was obtained. The grating period was reduced from 3.02 µm to 2.44 µm. Thus, a shrinkage of 19% was obtained.

## 4. Optofluidics

### 4.1. Microfluidics

Microfluidics describes the behavior of fluids through microchannels and the fabrication methods of microdevices that have channels and chambers in which liquids can flow [44]. Usually, femtoliters are used. The microfluidics field is very wide and comprises disciplines like physics, chemistry, electronics, materials science, biology, medicine and more [44,45,46,47,48].

Polymers, including silicone, are broadly used in the manufacture of microfluidic devices because they are robust and inexpensive while maintaining strong bio-chemical performance. Silicone allows rapid and effortless microfluidic chip fabrication. Furthermore, it has several advantages, such as its optical transparency, elasticity, low toxicity, chemical inertness, low cost and good gas permeability. Silicone can also be easily manipulated so that the fabrication of silicone devices may require few pieces of equipment. These properties make it particularly adapted for the fabrication of microdevices. As not all microfluidic structures work with light, we will focus on silicone devices that use light, this is, the optofluidics field. In the microfluidics field, PDMS has been used in the optical range and in other bands of the electromagnetic spectrum, such as terahertz [49,50].

### 4.2. Optofluidic Silicone Optical Elements

Optofluidics results from the combination of microphotonics, optics and microfluidics [51]. It uses light and liquids in miniaturized optical devices with lab-on-chip devices, fluid waveguides, deformable lenses, microdroplet lasers, displays, biosensors, optical switches and molecular imaging tools and energy [51,52,53,54,55].

#### 4.2.1. Optofluidic Lenses (Active Behavior)

**Figure 3:**
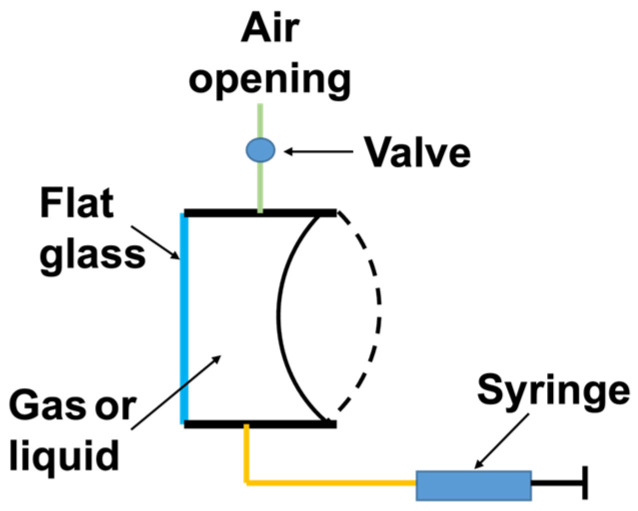
Bioinspired lens. The curvature of the lens changes (convex or concave) when pressure is modified.

Classical optical lenses made of hard materials do not change their focal distances, a characteristic that may be desirable in some applications. Optofluidic lenses can change the focal distance, allowing an amplified image. Animals, insects, and humans have developed compact, efficient optical systems throughout their evolution. Focal distances of animals’ eyes are modified when the curvature of the crystalline changes. At present, some instruments work with small lenses that change their focal distances by modifying the lens surface curvature. These are called bioinspired lenses. Several methods are used to change the curvature of lenses, such as electrowetting, dielectrophoresis, and surface tension. A bioinspired lens normally has a thin silicone membrane and a cell or chamber in which the fluid or gas is inserted. The silicone membrane profile may change when pressure is applied to the chamber. In general, by increasing the pressure, the membrane will become convex, resulting in a convex lens; by decreasing the pressure, a concave shape will be present, resulting in a concave lens (Figure 3). Some examples of different reported bioinspired lenses are cited in [56].

**Figure 4:**
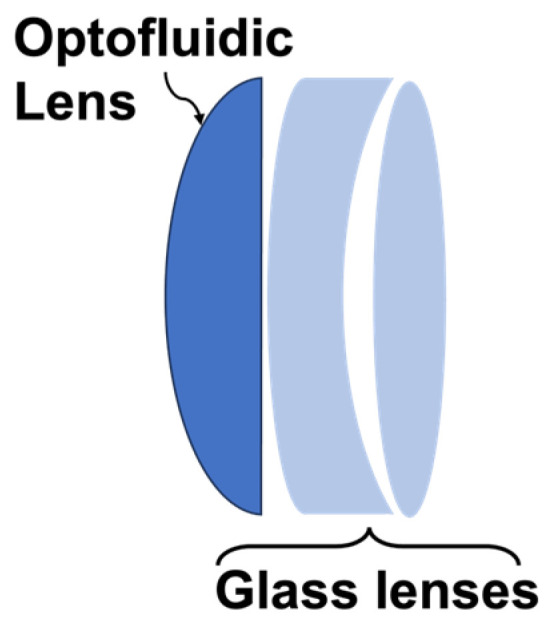
Optofluidic lens coupled with glass lenses.

However, bioinspired lenses consisting only of a silicone membrane normally present aberrations, such as field curvature. To correct these aberrations, glass lenses can be added. Thus, bioinspired lenses change their focal distances by changing the curvature of the membrane, and the glass lenses help to minimize the aberrations (Figure 4) [57].

**Figure 5:**
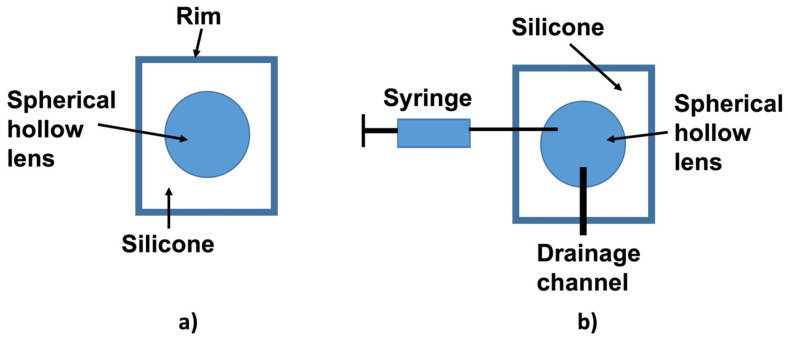
(**a**) Passive hollow microlens. (**b**) Dynamic microlens.

An approach for the fabrication of hollow microlenses [57,58] involved filling a plastic square rim (11 mm × 11 mm × 8 mm) with a mixture of silicone. The rim was used as a periphery structure (Figure 5). The bottom and top of the rim were sealed with thin (150 μm thickness) pieces of flat glass. Passive (Figure 5a) and dynamical hollow (Figure 5b) lenses were made by injecting air into the silicone mixture through a syringe, creating spherical lenses. The passive lenses could be filled with liquids that have one refractive index, creating a permanent focal distance, while in the dynamic lenses, it was possible to choose liquids with different refractive indices, allowing diverse focal distances with positive or negative values. The liquids employed in this work included ionic liquids, a class of organic salts [59]. The lenses were characterized for their focal distance and image formation [59].

**Figure 6:**
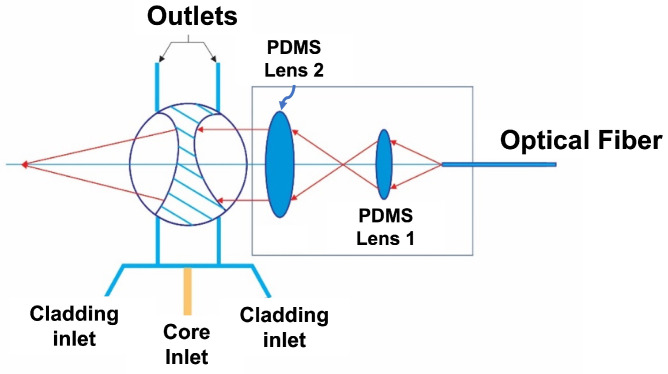
*Planar* optofluidic biconcave tunable lens. Liquids flow through the core (refractive index 1.36) and cladding (refractive index 1.54) inlets. Adapted with permission from [57]. © Optical Society of America.

An example of a *planar* biconcave micro-optofluidic lens was presented in reference [60]. The scheme of the optical liquid micro-setup is presented in Figure 6. In a silicone circular planar chamber, an optofluidic biconcave lens is formed in a liquid-core, liquid-cladding silicone structure. The liquids used to form the lens are ethanol (n = 1.36) and benzyl alcohol (n = 1.54). In the core channel, ethanol flows, and in the cladding channel, benzyl alcohol flows. Due to the shape of the liquids and their refractive indices, the fluidic setup behaves as a positive lens. The cladding flow rates are kept constant at 1 mL/h, while the flow rate of the core stream is adjusted to get different flow rate ratios Q_core_/Q_cladding_. Adjusting the flow rate ratio allows tuning of the focused beam.

#### 4.2.2. Optofluidic Gratings (Active Behavior)

**Figure 7:**
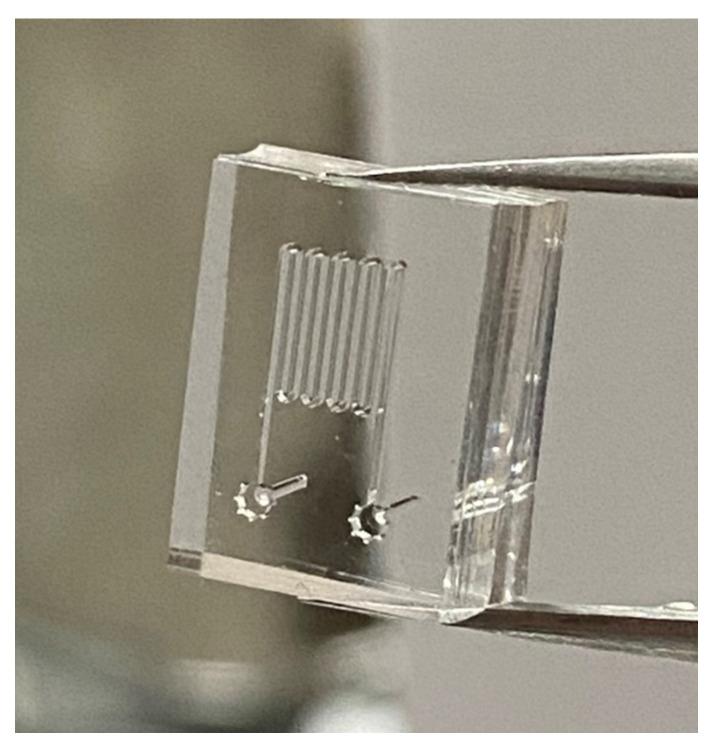
Optofluidic diffraction grating made in silicone.

Another optofluidic element is the optofluidic diffraction grating, which consists of an array of interconnected silicone microchannels [61]. This structure is in contact with a flat glass substrate. The microchannels can be filled with gas, aqueous solutions or organic solutions with a particular refractive index and a given absorption. The difference between the refractive index of the fluid and the silicone generates a phase difference in the light passing through the device, causing diffraction. Gratings can be reconfigured by filling the microchannels with different fluids. In the presented example, the microchannels have dimensions of about 200 µm wide and 500 µm deep. The fluid volume in the channels is about 10 nL (Figure 7).

#### 4.2.3. Optofluidic Lens with a Diffraction Grating on Its Convex Surface

**Figure 8:**
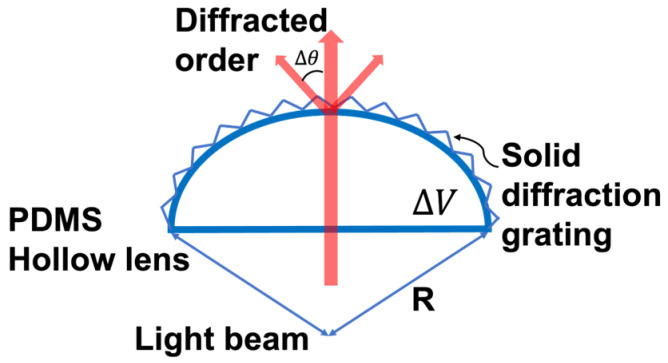
Optofluidic silicone lens with a diffraction grating on its curved surface. Adapted with permission from [60]. © Optical Society of America.

Another structure that comprises a hollow lens with a solid diffraction grating was reported in [62]. The lens focuses light, and the diffraction grating creates diffractive orders. The refractive index of the liquid in the lens determines the focal distance, and the period of the grating determines the angle of the diffracted light. The lens is a silicone hollow lens with a diffraction grating imprinted by soft lithography on one of its surfaces. The grating has 1200 lines/mm, with a triangular groove profile and a blaze wavelength of 240 nm. By injecting water, and thus increasing the pressure inside the lens, a distortion of the grating surface was achieved. He-Ne laser light was sent to the center of the lens, creating diffracted orders. The diffraction angle *θ* was monitored as a function of change in lens volume (Δ*V*). The fluid was injected with 0.1 mL increments, and total Δ*V* was limited to 1 mL. The diffracted beam spanned an angular range of Δ*θ* = 14.2°. The rate of beam deflection was dependent on the speed of fluid input, with no appreciable delay between volume expansion and the variation in the diffracted angle (Figure 8).

#### 4.2.4. Optofluidic Prisms (Active Behavior)

**Figure 9:**
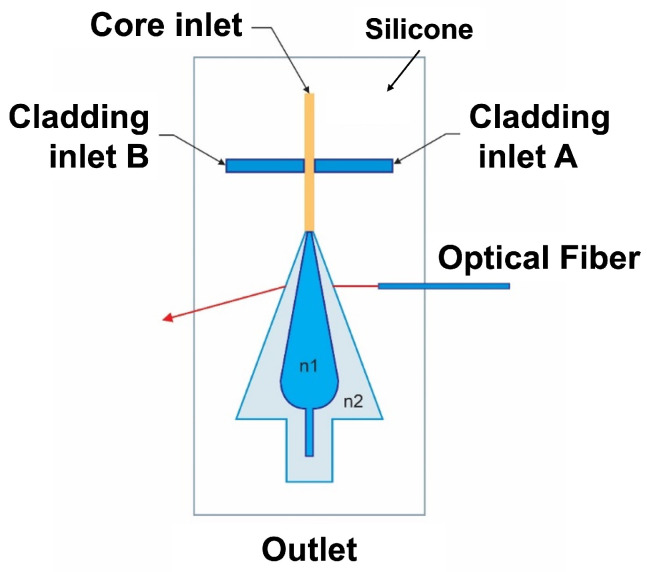
Optofluidic *planar* silicone prism formed by two liquids that are inserted from the core and the cladding. The prism is inside a silicone block. Adapted with permission from [61]. © Optical Society of America.

A tunable *planar* micro-optofluidic prism was hydrodynamically formed by one core and two cladding streams inside a sector silicone shaped chamber [63] (Figure 9). Cinnamaldehyde (viscosity = 5 × 10^−3^ Pas at 25 °C) with a refractive index of 1.536 was employed as the core stream, which formed the geometry of a triangular prism. A mixture (viscosity = 9 × 10^−3^ Pa s at 25 °C) of glycerol (60%wt) and water (40%wt) with a refractive index of n = 1.412, matching that of silicone, served as cladding streams. Owing to the higher refractive index of the core stream, this configuration can function as a prism. The apex angle of this optofluidic prism can be tuned by adjusting the flow rate ratio between the core and cladding streams; therefore, the deviation angle of an incident light beam can be changed accordingly. Since the propagation direction of a refracted light beam can be accurately controlled by choosing a proper flow rate ratio, this tunable prism can continuously scan the light beam and therefore can be used in the alignment of the optical path or in the development of optical switches.

Prisms can be used in spectroscopy to study beams with different wavelengths. Hollow prisms that can be filled with liquids with different refractive indices will change the beam path. The beam can be scanned when the liquid refractive index changes. Reference [64] shows that right-angle hollow prisms can be made using silicone. The dimensions of the reported prism were 7 mm, 7 mm and 10 mm. This prism can be filled with a liquid with a desired refractive index by means of a syringe.

#### 4.2.5. Optofluidic Variable Aperture (Active Behavior)

Common optical variable apertures used in optical laboratories consist of steel blades that are linked by sliding a rotary knob, creating a variable transparent polygon aperture. However, this complicated structure limits miniaturization and the application areas. Fluidic aperture designs have been developed to overcome these limitations.

**Figure 10:**
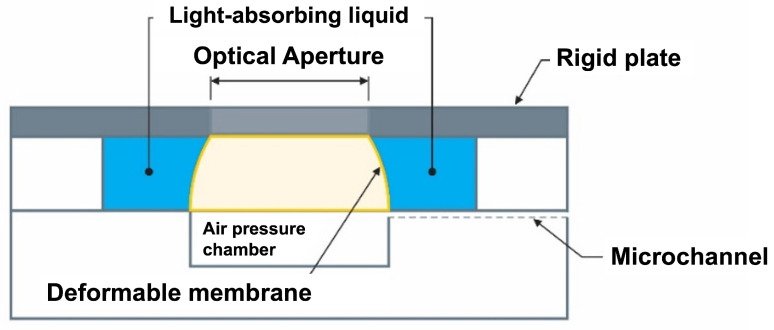
Optofluidic variable aperture in silicone block. Adapted with permission from [63]. © Optical Society of America.

An optofluidic aperture that shows a change in diameter from zero to 6.3 mm was presented in reference [65]. The aperture has a nearly circular shape with a continuously adjustable diameter. The device consists of a cavity that is filled with a light-absorbing liquid between a rigid transparent upper plate and a thin circular membrane made of silicone. An air pressure chamber and a microchannel control the membrane size. When increasing the air pressure, the membrane shows a convex profile. When pressure increases, the deformation of the membrane also increases, and the light-absorbing liquid is replaced by air, forming a circle and allowing light to be transmitted through this region. Thus, the diameter of the optical aperture can be continuously changed by inflating or deflating the air pressure chamber (Figure 10).

## 5. Optical Devices Made with Silicone Optical Components

### 5.1. Refractometers (Active Behavior)

Classical refractometers measure the angle of refraction at the liquid–solid phase interface [66]. A quick evaluation of the concentration of dissolved substances can be made using the angle of refraction, which depends on the composition of the solution. The Abbe and Pulfrich refractometers are two prism-based refractometers based on this principle.

**Figure 11:**
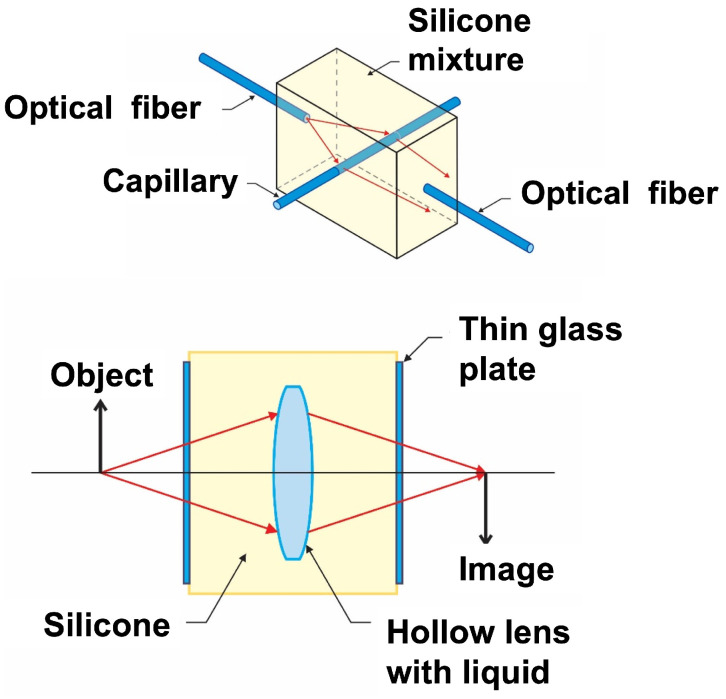
Optofluidic refractometers using PDMS. A capillary is immersed in a silicone block. Through this capillary, the liquid to which the refractive index is measured is inserted. Optofluidic refractometer composed of a hollow lens inside a silicone block. By changing the liquid in the lens, the distance of the image from the lens changes. Adapted with permission from [68]. © Optical Society of America.

However, refractometers based on different principles have been developed over time. One of these new refractometers is based on a capillary that is embedded in silicone [67]. Another two examples are based on silicone diffraction gratings [68,69], and another uses a silicone optofluidic lens [70]. These systems are depicted in Figure 11. Finally, a refractometer based on tapered optical fibers immersed in silicone was presented in [71].

### 5.2. Pressure Sensors (Active Behavior)

Pressure is defined as the amount of force exerted by a gas or liquid applied to a unit area [72]. Pressure sensors are built with different technologies, designs, performances, stabilities and costs. Pressure sensors find a wide range of applications in industry, medicine, semiconductor processing, environmental applications, manufacturing processes, automotive and more. We next describe some optofluidic pressure instruments based on membranes and diaphragms made with silicone.

#### 5.2.1. Deformable Diffraction Grating (Pressure Sensor) (Active Behavior)

An optofluidic diffraction grating was proposed to monitor the local pressure in a microfluidic device [73]. The grating was molded in silicone covering an area of 2 mm × 2 mm, with rectangular grooves 5 microns wide and 2 microns deep and a period of 10 microns. All the grooves were connected to a microchannel 200 microns wide and 20 microns deep. The grooves were deformed through this microchannel by applying internal pressure. As pressure was applied, the diffracted orders showed different angular positions. Measured pressure values were between −80 kPa and 100 kPa. Moreover, the device could be used to measure air flow, with rates ranging from 0 to 0.3 mL/min.

#### 5.2.2. Fabry–Perot Interferometer Used as a Low-Pressure Sensor (Active Behavior)

**Figure 12:**
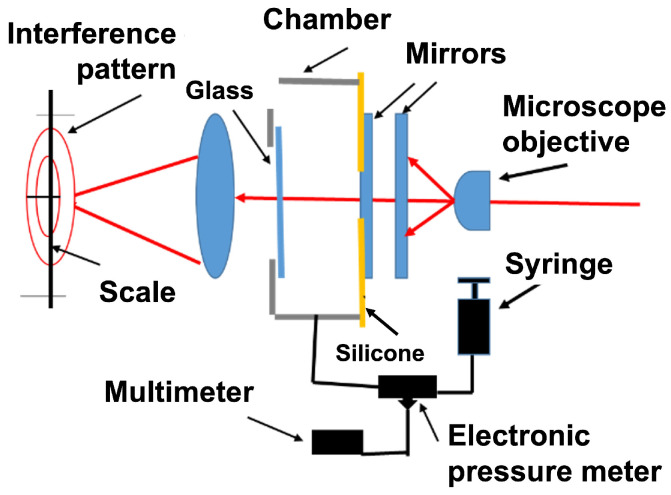
Fabry–Perot interferometer used to measure low pressures. One mirror is glued to a silicone membrane to allow longitudinal displacement when the pressure changes. Adapted with permission from [74].

A Fabry–Perot device for low-pressure measurements comprises a metallic cylindrical chamber with a silicone transparent flexible thin membrane and a high-reflectance mirror glued to one of its ends [74]. The silicone membrane thickness was 546 µm. To form the optical Fabry–Perot cavity, another mirror was placed in front of the first one (Figure 12). 

After the light crosses the optical cavity, an interference pattern, consisting of a series of concentric light rings, is formed by the lens. When the pressure increases/decreases in the chamber, the glued mirror will undergo linear displacement, and the rings will show radial displacement according to the pressure change. The independent variable for the calibration curve is the pressure, and the light fringe displacement is the dependent variable. The displacement was measured with a reticle (scale). This method measured low-pressure values on the order of tens of Pascals with a sensitivity of 3.3 mm/Pascal. Better sensitivity was obtained when the distance between mirrors was on the order of a few millimeters.

#### 5.2.3. Plastic Optical Fibers on a Silicone Membrane (Pressure Sensor, Active Behavior)

This pressure sensor consists of two PMMA optical fibers and a flat silicone membrane with a thickness of 8 µm [75]. One light-conductive fiber is fixed over the silicone surface membrane and the second fiber crosses over the first one at 90°. A single pressure point at the intersection of both fibers is obtained. When pressure is applied to the membrane, a mechanical deformation at the first fiber results in a change in the amount of light traveling through the second optical fiber. Some energy may be lost due to light dispersion. A detector measures the change in light intensity suffered by the first fiber to calculate the pressure exerted on the membrane. Pressures from 63 kPa to 509 kPa were measured.

#### 5.2.4. Pressure Sensor with an Optofluidic Lens (Active Behavior)

**Figure 13:**
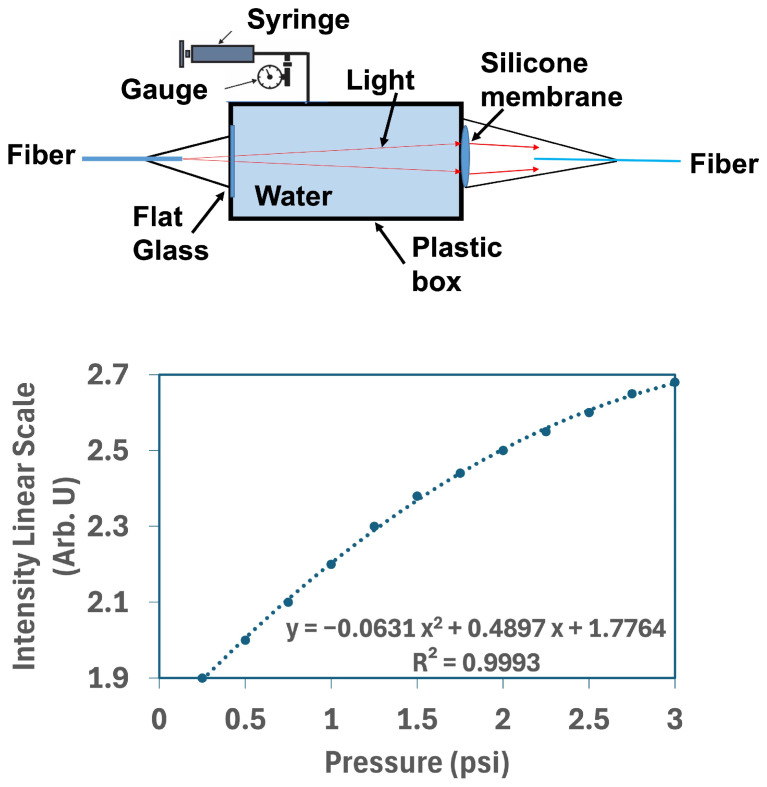
Pressure device with a silicone membrane and calibration curve for the pressure sensor. The quadratic equation of the best-fitting curve is shown, with the corresponding R value. The points refer to experimental data, while the dotted line is the fitting line. Adapted with permission from [75]. © Optical Society of America.

The sensor suggested in reference [76] consists of two fibers and a deformable membrane that forms a positive lens under pressure. The optical configuration in Figure 13 shows the location of the different elements that compose the pressure sensor. The cell was made with a structured square plastic rim. Two circular apertures of about 3 mm were drilled, one on each side. A thin glass window was mounted on one aperture, and a thin silicone membrane with a 1 mm thickness was attached on the opposite side. Light was sent to the cell by means of an SM 600 optical fiber, and the focused light was collected with a 100/125 fiber. When the pressure in the cell was increased by means of a syringe, the thin silicone membrane formed a positive lens, focusing the light. The second fiber collected the light and sent it to a detector. The calibration plot of collected intensity as a function of pressure was obtained. Values between 0.5 psi and 3 psi were measured (1 psi = 66.8 kPa).

#### 5.2.5. Pressure Measurements Through Image Analysis (Active Behavior)

**Figure 14:**
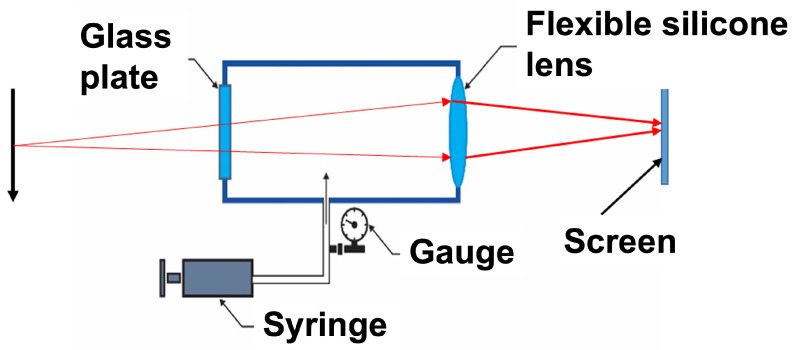
Optofluidic configuration used to measure pressure by modifying the curvature of a silicone lens by applying pressure. Adapted with permission from [76]. © Optical Society of America.

In reference [77], a method for measuring pressure through images formed by a flexible optofluidic silicone lens is shown. The visibility of the images formed by the lens on a screen changes when the pressure is increased or decreased (Figure 14). The structure of the detector consists of an acrylic tube with a flexible silicone lens mounted on one end and a flat-plane glass attached to the opposite end. On the periphery of the tube, a screw with a hole in its central part is fixed. The pressure inside the tube can be changed by injecting air through this hole with a syringe. This pressure change modifies the curvature of the lens. A calibration plot relates the visibility of the images to pressure. A pressure value from 0.1 psi to 0.8 psi was measured (1 psi = 66.8 kPa). This method can be used for other pressure values by changing the lens curvature, refractive index, or stiffness and size of the lens.

#### 5.2.6. Monitoring Volatile Organic Compounds with a PDMS Surface Grating (Active Behavior)

Volatile organic compounds (VOC) are harmful compounds present in building materials, care solutions, aerosols, and paints, just to mention a few. VOC detection methods include MEMS systems, gas chromatography, photoionization detection and more. Among transducers, PDMS was used for optical VOC detectors like distributed Bragg reflectors, fiber Bragg gratings and more. The VOC PDMS transducer swells or changes its refractive index when a VOC permeates it, allowing the gas concentration to be measured.

In reference [78], a VOC detector based on a PDMS surface diffraction grating is shown. Several liquids were tested, such as toluene, m-xylene, chloroform, n-butanol and ethanol. To make the PDMS grating, a master surface grating was first inscribed in an acrylamide film by recording a two-beam interference pattern. Then, an unpolymerized mixture of PDMS was poured over the acrylamide surface grating to make a copy of the master grating. After polymerization, the PDMS film was peeled off from the master grating and placed over a flat glass. Gratings with periods from 2 to 8 µm and surface relief depths between 120 and 530 nm were used for investigations. The profiles of the gratings’ surfaces were characterized with an AFM microscope.

**Figure 15:**
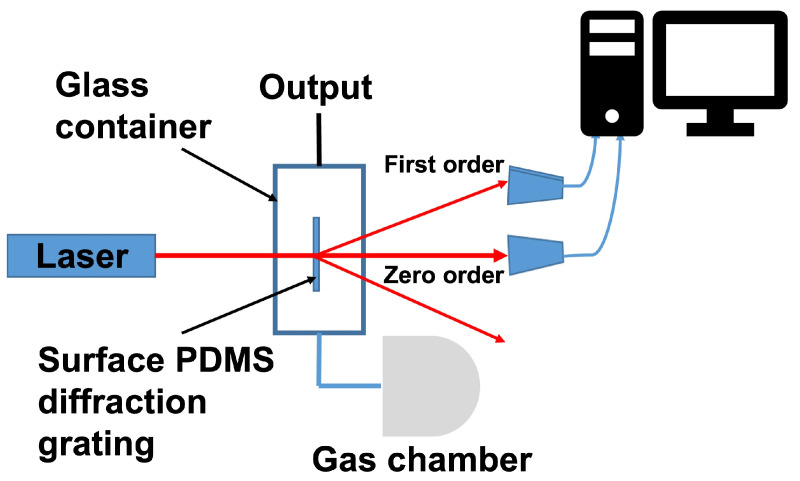
Optical configuration for monitoring VOC by means of a PDMS surface diffraction grating. Adapted with permission from [77].

The optical configuration to detect a VOC is shown in Figure 15. A gas chamber with an analyte is connected to a glass container with the PDMS grating. A laser illuminates the grating, which diffracts light into three diffraction orders: −1, 0 and +1. Detectors collect the intensity of the zero and +1 order. Liquid concentrations were calculated using a known concentration of air (0.294), liquid volume, liquid density and liquid molecular weight, M (g mol^−1^). Finally, a plot relating the first-order intensity to the concentration was obtained.

#### 5.2.7. Biomedical Optical Integration

##### Optofluidic Microscope (OFM, Active Behavior)

**Figure 16:**
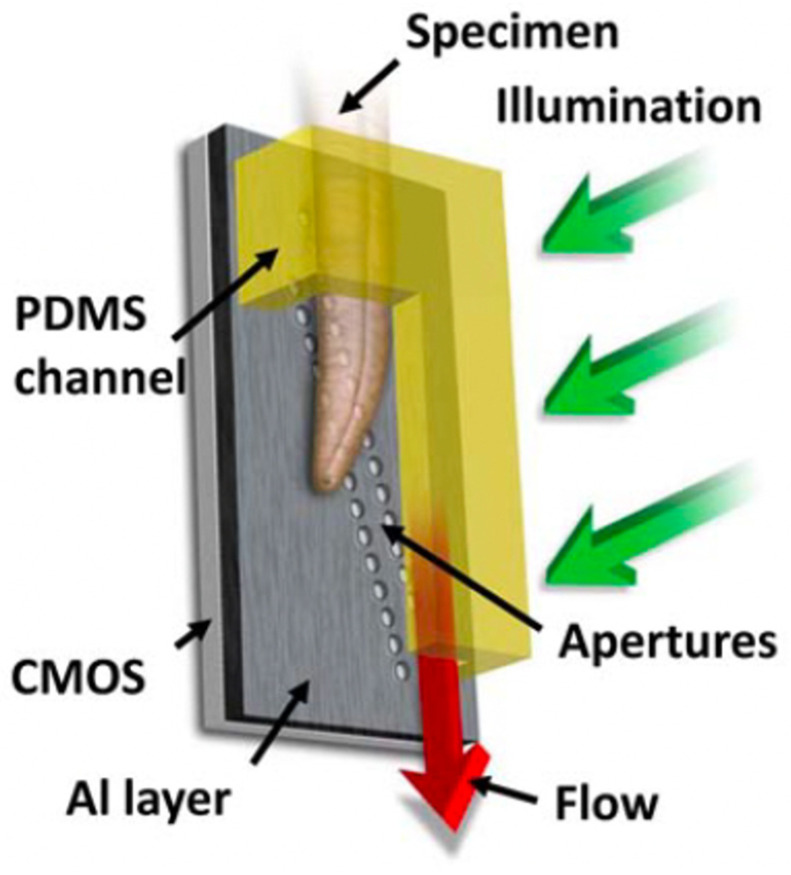
Configuration of an optofluidic microscope to scan a *C. elegans* worm. The green arrows represent the illumination of the device, and the red arrow shows the direction of the flow within the channel. Reproduced with permission from Cui, X, et al., *Proceedings of the National Academy of Sciences of the United States of America*, Vol 105, pp. 10670–10675, (2008) [61] Copyright (2008). National Academy of Sciences.

The OFM consists of a C-MOS light sensor (9.9 µm × 9.9 µm pixel sensor size) coated on its surface with a thin aluminum metal film (300 nm thick), which has apertures placed in a row (1 µm diameter, spaced 9.9 µm) etched on its surface [79]. Over this metallic layer, a silicone structure with a microfluidic channel for a sample is placed. In one work, a *C. elegans* worm was studied. On top of these elements, a halogen source (20 mW/cm^2^) is placed. To complete the scanning, the *C. elegans* worm flows through the channel, and the line scans are electronically acquired (Figure 16).

##### Measurements of Artery Pulse Waveform (Active Behavior)

**Figure 17:**
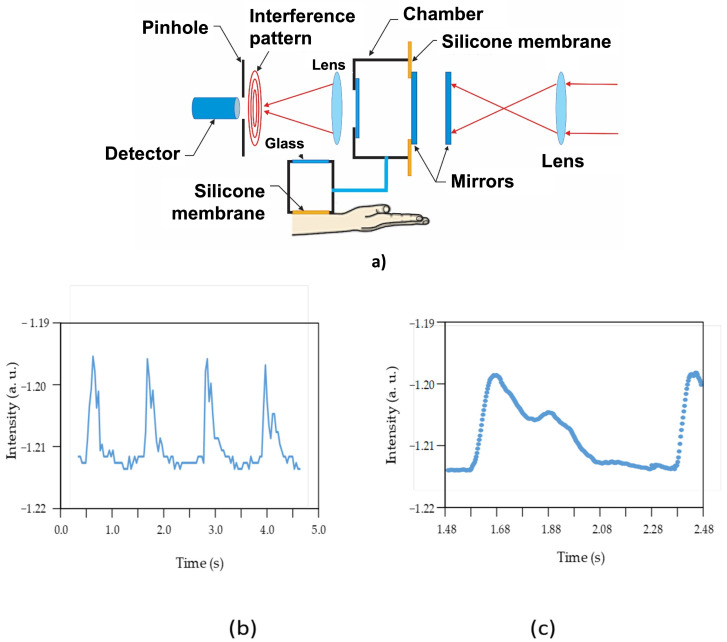
(**a**) Scheme showing the Fabry–Perot interferometer applied to measure the artery pulse waveform. (**b**) Plot of artery pulse waveform. (**c**) Waveform corresponding to one artery pulse. Adapted with permission from [80].

Another application of the Fabry–Perot interferometer that uses a silicone membrane is the measurement of arterial pulse pressure. The setup used to measure arterial pulse pressure is shown in Figure 17a [80].

Light from a He-Ne laser is sent to a microscope objective and then to the Fabry–Perot cavity comprising two high-reflectance mirrors. One mirror is fixed, and the other is attached to a thin silicone membrane supported by a circular aluminum cell. After the light traverses both mirrors, a lens collects the light, and an interference pattern consisting of concentric circles is formed at its focal distance. The circular aluminum cell is connected to an acrylic chamber by a hose. The acrylic chamber has a thin silicone membrane and a flat glass at opposing ends. The silicone membrane is placed on the wrist artery. When a blood pulse stretches the membrane, an air pulse is sent to the metallic cell in the Fabry–Perot cavity, and the mirror at the silicone membrane moves axially, causing displacement of the fringes in the interference pattern. This variation is collected by a detector and relates to the arterial pulse pressure (Figure 17b,c).

## 6. Photothermal Detectors (Active Behavior)

As silicone has a thermal expansion coefficient of 3 × 10^−4^ cm/°C, it may be used for the design of photothermal detectors. For example, a device with a surface relief grating on a 1 mm thick slab of silicone was used [81]. This grating was dyed with a mixture of ethanol:hexane (3:2) solution saturated with *Sudan red* for 30 min. Then, the relief grating was heated with light from an argon laser with a wavelength of 514 nm. The lateral thermal expansion induced on the slab by absorption of green light changes the pitch of the grating. Light from a He-Ne laser (638.2 nm) was overlapped with the heating beam on the grating sample so that the expansion induced by the argon beam was detected by measurement of the angular deflection of the diffracted beam. The angular deflection was measured as a function of the intensity of the heating beam. Response times on the order of 25 s were found. The sensitivity limit of this detector is on the order of a few μW/mm^2^.

**Figure 18:**
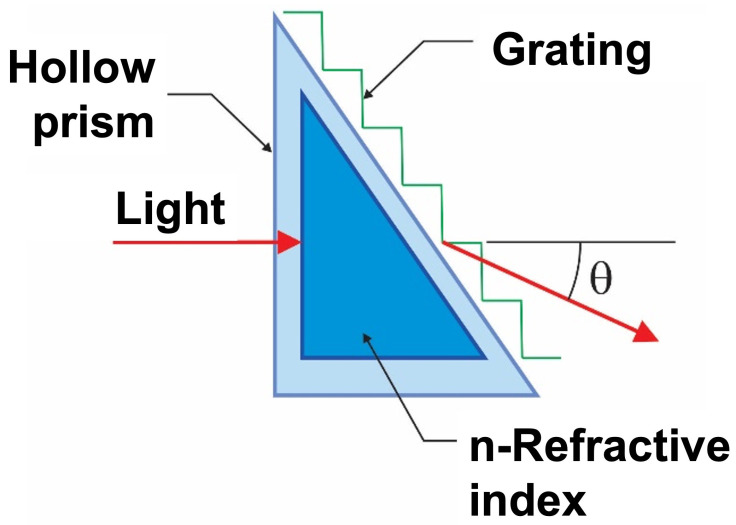
Optofluidic configuration to measure temperature. A hollow silicone prism with a grating on one of its sides. Adapted with permission from [83].

Another method for measuring temperature was done with a hollow grism. A solid grism keeps light with a given wavelength undeviated as it passes through it, as shown in Figure 18. However, a solid grism is designed for a specific wavelength that cannot be changed after its fabrication. Thus, an optofluidic grism can be used to modify the useful wavelength of the grism. A combination of a silicone optofluidic prism and a silicone solid blaze grating was implemented to make an optofluidic grism. The silicone solid blaze grating was placed at the hypotenuse of the right-angle prism. The elastomeric blaze grating was made by casting a silicone prepolymer on a master grating with a pitch of 1.68 µm and a groove angle of 8° [82]. When the temperature of the liquid inside the prism changes, the angular deviation of the light emerging from the grism will change. A calibration plot can be made for the temperature as a function of the angular deviation of light.

## 7. Silicone Polarization Devices (Active Behavior)

**Figure 19:**
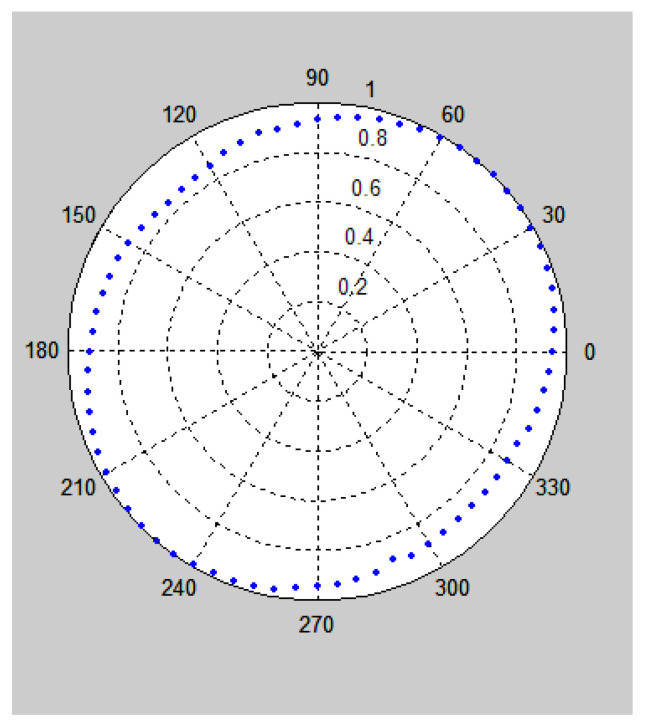
Behavior of first-order light intensity, radial coordinate, as a function of analyzer angle. The blue dots represent the experimental data. Adapted with permission from [81]. © Optical Society of America.

In Section 4.2.2, we described optofluidic gratings where liquids flow. In addition to this, it is possible to insert anisotropic liquids into optofluidic gratings [83]. One of these liquids is penicillin. Due to its chirality, the liquid has a circular birefringence of 2.14 × 10^−7^. The addition of the anisotropic liquid to microchannels modified the polarization properties of diffracted orders. The diffraction efficiency of the grating was characterized for different probe beam wavelengths and states of polarization. The optofluidic grating had channels with a width of 200 μm, a distance between them of 200 μm and a depth of 500 μm. The channels were 5 mm long with a square profile. In a polar coordinate system, the radial coordinate is the first-order intensity, and the analyzer angle is the angle. They were monitored when the microfluidic grating had a mixture of penicillin/water. Incoming or reading light (λ = 632.8 nm) was circularly polarized. Figure 19 shows the first-order intensity, where it is possible to notice that the first order also showed a circular polarization.

## 8. Silicone Waveguides

Optical waveguides are important in optical detection. These waveguides confine and transport light in a micro-chip, direct light to the sample confined in a micro-volume and collect the emitted radiation for detection analysis.

There are several types of optical waveguides, and silicone is one of the materials used to fabricate planar waveguides because its elastomeric properties present optical transparency and low attenuation. Planar waveguides have a high-index strip where the light is guided (core). This high-index layer is confined between two low-refractive-index layers called cladding. Various structures of waveguides can be found, for example, solid-core/solid-cladding, liquid-core/solid-cladding, liquid-core/liquid-cladding or hybrid [84]. 

An all-silicone waveguide comprising core and cladding was developed. The silicone refractive index was changed by setting different mixing ratios between the base and the curing agent [84]; thus, silicone can be used in the fabrication of the core and cladding. The transmission loss of the fabricated all-silicone waveguides was about 1.1 dB/cm at 460 nm wavelength.

Other attempts have also been made to make silicone waveguides. Polymeric materials are low-cost, the process is done at room temperature, and they have design flexibility. Because light travels very short distances in telecom devices, polymers can be used in Datacom chips. Silicones have passed the tests (Telcordia) in the fabrication of waveguides. Silicone waveguides present good thermal and optical stability, durability, and thermo-optic coefficients (dn/dT) and acceptable losses over short distances [85,86]. The production of waveguides from silicone film by photopatterning using UV light was shown. Fabricated waveguides 50 µm wide and 50 µm high, with minimal wall roughness, were achieved.

## 9. Silicone Cytometers (Active Behavior)

**Figure 20:**
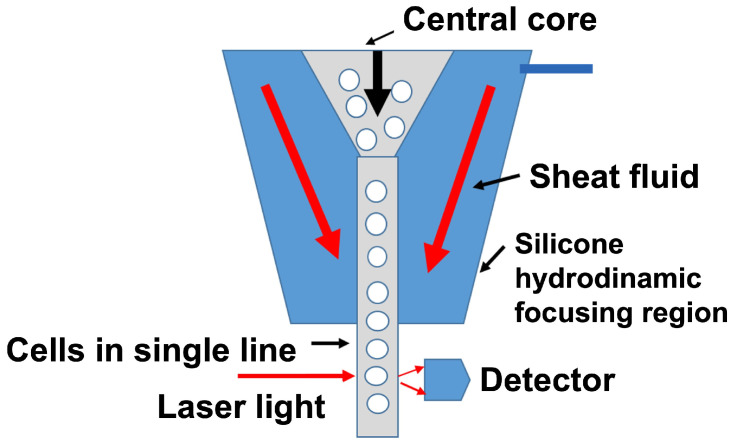
A single stream of particles is produced by hydrodynamical focusing. Then, laser light illuminates each particle. Light is collected by a detector. The red arrows within the chamber represent the direction of the envelope fluid.

The definition of cytometry could be the ability to measure properties of individual particles as they flow through a silicone microchannel. Cytometers were developed in the first half of the last century [87,88]. Nowadays [89], cytometers mainly comprise the following parts: fluidic system, optics and detection, signal and pulse processing, and electrostatic cell sorting, among others. The fluidic system (Figure 20) consists of a central core through which the sample fluid is injected, enclosed by an outer envelope fluid. Due to the narrowing of the envelope (in a silicone nozzle or cuvette), the fluid velocity is increased. A sample is introduced into the central fluid and is focused. This allows the creation of a stream of particles in a single row and is called *hydrodynamic focusing*. There is no mixing of the central fluid stream and the envelope fluid. The fluidics system can be made with several materials, like glass, silicone and others [90]. The sample stream has a diameter of roughly 5 microns, and velocities through the channel can be up to 10 m/s. When cells or particles travel through the nozzle of the instrument, they are illuminated with a focused light, typically a laser light. The particles scatter light and undergo fluorescence emission, providing information about the particles’ properties. Light that is scattered in the forward direction after being transmitted by the particles is collected by a photomultiplier or a photodiode. This step is known as the *forward scatter channel*. This will give an estimate of the particle size. However, another channel that collects the light that is scattered at a 90° angle to the excitation line is the *side scatter* channel. This channel gives information about the relative complexity (granularity and internal structure) of a cell and a particle. Furthermore, using multiple interrogation points with different lasers, it is possible to collect as many as 19 optical parameters from a single particle. A helpful review of cytometry can be found in reference [91].

## 10. Silicone in Light Sources (Passive Behavior)

Silicone has also been used in Solid-State lighting (SSL) sources (LED) [92,93]. The structure of a basic LED comprises a chip where blue light originates (450 nm). This LED is covered by a silicone encapsulant to prevent it from being in contact with environmental stresses. In addition, a silicone lens containing phosphor particles is placed on top of the chip. The phosphor converts blue light into red light (650 nm). The combination of blue and red light gives white light.

In the case of electrical overstress, silicone materials carbonize, resulting in dielectric breakdown. Silicone carbonization can be caused by over-absorption of blue light by the phosphor, resulting in joule heating and causing degradation of silicone.

## 11. Fiber Bragg Grating Wearable Sensor (Passive Behavior)

There are illnesses, like Strokes, that cause post-Stroke symptoms like facial paralysis, speech disorders and others. There is a need to rehabilitate fine motor skills, such as wrist, finger and mouth movements. Thus, the use of cost-effective, portable, flexible and comfortable wearable sensors for long-term use in personalized health care should be developed. One of these wearables uses Fiber Bragg Gratings (FBGs). FBGs can measure strain, pressure, angle, torque and other values, to mention but a few. An effort was made to fabricate FBG wearable sensors with robustness, adaptability, and sensitivity by embedding an FBG in silicone [94]. This reported wearable consists of an FBG embedded in a silicone patch. The FBG length was 10 mm and used light with a wavelength of 1550 nm. The patch dimensions were 40 mm by 20 mm, and thicknesses of 1 mm, 2 mm and 3 mm were tested. The FBG was embedded in these patches, and the output spectra of the fiber were analyzed by a Bragg meter. Experiments included wrist pitch monitoring, finger joint movements and gesture recognition (mouth movements).

## 12. Joule Heating-Induced Carbonization Process of PDMS

The thermal degradation of linear PDMS was studied under two heating regimes: a slow, temperature-controlled process and a rapid heating process via flash pyrolysis [95].

During thermal degradation under slow heating in vacuum, molecular scission of the Si–O bonds in the PDMS main chain occurs. The resulting products consist of a coexistence of cyclic PDMS oligomers—formed through structural rearrangement of cleaved molecular segments—and linear PDMS chains that have not yet undergone bond scission.

In other words, a fraction of the linear PDMS molecular chains break into smaller fragments; however, instead of remaining linear, these fragments undergo structural rearrangements to form cyclic, crosslinked molecular structures [96], as illustrated below:

$${\mathrm{PDMS}}_{\mathrm{linear}}⟶{\mathrm{SiO}}_{x}C_{y\;\left(\mathrm{Crosslinked}\;\mathrm{cyclic}\right)}+{\mathrm{PDMS}}_{(\mathrm{Residual}\;\mathrm{linear})}$$

Through rapid thermal degradation (flash pyrolysis at T = 800–1000 °C), linear PDMS decomposes into crosslinked cyclic oligomers.

Simultaneously, these crosslinked cyclic PDMS oligomers undergo homolytic cleavage of the Si–CH_3_ bonds, releasing hydrogen and forming methane gas (CH_4_) [97]. In other words,

$${\mathrm{PDMS}}_{\mathrm{linear}}⟶{\mathrm{SiO}}_{x}C_{y\;\left(\mathrm{Crosslinked}\;\mathrm{cyclic}\right)}+{\mathrm{CH}}_{4}$$

In addition to thermal degradation processes, there is an ultrafast PDMS carbonization process known as Joule heating of phosphorus particles. This process involves applying a high electrical potential to PDMS (a dielectric polymer). As a result, PDMS undergoes near-instantaneous carbonization, as its molecular structure experiences internal chemical transformations, converting it into a highly conductive material.

It is important to note that the chemical mechanism underlying PDMS carbonization via Joule heating is not yet well understood. However, it is believed that the ultrafast thermal shock induced during Joule heating promotes the formation of PDMS’s carbonized phase. Thus, the Joule heating process effectively bypasses the intermediate chemical species typically formed in other heating systems [97,98].

In our view, the carbonization of PDMS resulting from Joule heating of phosphorus particles may occur as follows:

$${\mathrm{PDMS}}_{\mathrm{linear}}⟶\underset{Intermediate\;\;stage\;\left(∼1400\;{}^{\circ}C\right)\;eliminated\;}{\underbrace{{\mathrm{SiO}}_{x}C_{y\;(Crosslinked\;cyclic)}+CH_{4}}}⟶\underset{\mathrm{Final}\;\mathrm{stage}\;\geq \;1700\;{}^{\circ}C}{\underbrace{{\mathrm{SiC}}_{\left(Crosslinked\;solid\right)}+C}}$$

Therefore, the carbonization of PDMS via Joule heating of phosphorus particles may proceed as follows:

$${\mathrm{PDMS}}_{\mathrm{linear}}⟶{\mathrm{SiC}}_{\;\left(\mathrm{Crosslinked}\;\mathrm{solid}\right)}+C$$

And schematically, it could be represented as follows:

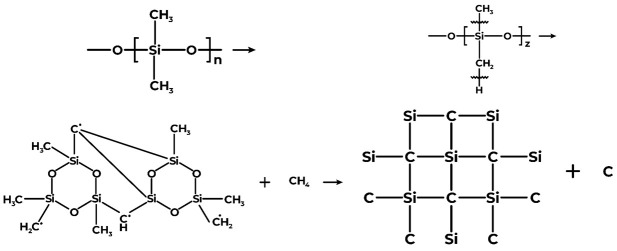



## 13. Other References: Silicone Applications in Other Fields, Journals and Books

We would also like to highlight some other interesting applications of PDMS outside the field of optics. Even though the scope of this work is within this field, we highlight that there are other promising applications in technology development that are worth mentioning. First, PDMS has been used in the field of wearable skin-inspired electronics. Another method [99] for fabricating a master microfluidic channel mold involves using Kodak FLEXCEL NX Ultra Solution and then using silicone to make the microfluidic channel. From a single master mold, about 50 silicone copies can be made with no degradation of the mold. Channel heights between 53 microns and 1500 microns can be made. Silicone wearables have been used in stretchable, durable health sensors, strain-insensitive pressure sensors (sensitivity of ~83 kPa^−1^ and 5000 durable cycles), robust alternating current electroluminescent displays, and flexible organic light-emitting diodes (20% improved luminescent and 300 flex endurance of 2 mm bend radius) [99]. Second, light-emitting copolymers have been developed by incorporating diarylfluorene-based trimer and polydimethylsiloxane (PDMS content < 30%), with precisely tunable mechanical properties from brittleness to viscoelasticity [100].

Polysiloxanes and their derivatives may also be modified to present the interesting characteristic of self-healing. This is the ability of a material to restore its initial characteristics after some damage. Reviews of self-healing polysiloxane-based materials and their applications have been recently published [101,102]. PDMS can activate repair mechanisms in response to mechanical or electrical damage, preventing failure. These materials can show their states through color changes, fluorescence, or luminescence when an external stimulus affects them. They can eliminate contaminants and ice films from their surfaces [103]. Optoelectronic applications such as liquid crystal devices, memory drives, OLED, photovoltaic and optical waveguides are described in these reviews. The novelty of some of these applications relies on creating flexible devices with self-healing silicone materials. A PDMS elastomer designed for smart sensors showed rapid self-healing capabilities under both UV and visible light irradiation with high self-healing efficiency, achieving 98% strength recovery within 30 min under UV light and 97% recovery within 60 min under xenon light [104]. Furthermore, a self-healing polydimethylsiloxane (PDMS, Fe-HPDCA-PDMS) and carbon nanotube composite was reported as a flexible optoacoustic patch. This device can recover from damage induced by cutting or laser irradiation, and it was successfully examined in acoustic flow, thrombolysis, and wireless energy harvesting applications [105].

An innovative C-PBS/CNT composite with high stretchability, high adhesion, and multichannel recording capabilities was reported in [106]. This modified material was reported as a significant advancement in polymer-based bio-signal interfaces for robust, long-term solutions in epilepsy research and clinical applications, overcoming some of the problems with PDMS in clinical applications such as poor adhesion to skin and excessive stiffness of tissue interfaces. A real-time glucose detection film has been fabricated by using a photonic crystal PC-PDMS array. Polymethyl methacrylate PCs are deposited onto a substrate composed of a PDMS–glass slice with hydrophobic surfaces. This substrate can reflect blue light while allowing other colors to pass through, improving glucose sensitivity [107].

There are also extended reviews and reports on the use of PDMS in the fields of optical communications and Internet of Things that can be found in references [108,109,110,111,112].

Finally, human skin-inspired electrospun robust strain-insensitive pressure sensors and wearable flexible light-emitting diodes have been developed [113].

The previous descriptions are evidence of the versatility of PDMS not only in optics but also in other technological fields such as electronics and biomedicine.

## 14. General Remarks

The applications presented in this manuscript give a general idea of the versatility of using this material in optical applications. The presented review is intended as an overview and does not aim to provide exhaustive quantitative comparisons with other useful materials such as glass, PMMA, and hydrogels. These materials are also widely used in optics, and each of them presents different optical, mechanical and chemical properties. PDMS shares some qualities with these varied materials, but it also has inherent differences. Factors such as optical performance, flexibility, fabrication complexity, environmental stability, and biocompatibility will identify the best candidate for each application. Throughout this review, we have highlighted the qualities and advantages that silicone may present compared to the other existing options for a specific application. Future work may include a systematic quantitative analysis to establish standardized performance criteria for PDMS against all other potential options in optical applications. In general, PDMS has high flexibility, deformability, and microfabrication compatibility, which makes it especially valuable in wearable optics and optofluidics.

To extend the information presented in this brief review, a few journals and books in which silicone and light are more often cited are presented in references [114,115,116,117,118,119,120,121].

## 15. Companies That Fabricate Silicone Components

Finally, we refer to a company [122] that fabricates silicone optics that work in extreme environments. These optics are resistant to temperature, UV light, and humidity and can be used with UV, visible and NIR light (350 nm–1600 nm) because they transmit nearly 100% at those wavelengths.

## 16. Conclusions

Polydimethylsiloxane (PDMS or silicone) is a polymer with interesting and optimal chemical, optical and physical characteristics that are dependent on the degree of polymerization and the R and R’ groups, as well as on the molecular crosslinking density. Some of the main characteristics of PDMS are flexibility due to oxygen–silicon bonds, hydrophobicity that may be modified by treatments involving grafting other polymers into the silicone matrix and high transparency and low scattering and absorption in the UV and visible ranges. These last features allow the application of PDMS particularly in the field of optics. In this review, we presented some relevant uses of PDMS in optics. Microlenses and microlens arrays have been fabricated with diameters of 700 to 100 microns; reported diffraction gratings have spatial frequencies ranging from 3.5 to 14 lines/mm; and hybrid optics combining lenses and diffraction gratings can also be fabricated. In the field of opto-microfluidics, PDMS has also been used to fabricate optical elements and lab-on-chip devices. Bioinspired lenses, passive and dynamic microlenses, and optofluidic diffraction gratings, among others, have been described. We have also reviewed different optical devices based on PDMS components. Refractometers, pressure sensors, artery-pulse sensors, VOC sensors, microscopes, thermal detectors, polarization devices, waveguides and cytometers are just a few examples of PDMS applications in the field of optics. The uses of PDMS in optics also overlap with other technological areas such as electronics. Due to its flexibility and optical transparency, PDMS has been used for LED and OLED fabrication and for the development of wearable and self-healable devices. Due to its mechanical deformation, it may continue to help the development of applications such as flexible photonic circuits and strain-sensitive optical systems. The characteristics of PDMS that may limit its applications are mechanical aging, solvent absorption, hydrophobicity, and refractive index contrast. However, PDMS has proven valuable in applications that require optical flexibility and where conventional optics are unsuitable. Furthermore, silicone can be improved by forming silicone composites that may exhibit new properties such as self-cleaning, self-healing and hydrophobicity. These silicone composites can be used for new optical devices and may be applied in areas such as biomedical, electronics, microfluidics, membranes, sensors (including wearable), coatings, energy harvesting and wastewater systems, injection molding, Non-Contact Fiber (NCF) optics interferometers for coating measurement, optical fiber coatings and more.

## Figures and Tables

**Table 1:** Common reported properties of cured PDMS polymers.

Parameter	Unit	Value	Observations	Reference
Optical Properties				
Transmittance	%	75–92	From 390 to 780 nm	[11,22]
Refractive index of PDMS Refractive index of PDMSDPS ^†^		1.41 1.43	589 nm 589 nm	[4,6] [6]
Electrical Properties				
Electric conductivity	Ω/m	4 × 10^13^		[23]
Dielectric constant	F/m	2.3–2.8		[23]
Dielectric strength	V/µm	14		[24]
Volumetric resistivity	Ω/cm	2.9 × 10^14^		[25]
Thermal Properties				
Thermal conductivity (PDMS)	W/m·K	0.2–0.27	At 27 °C	[25,26,27]
Thermal conductivity of PDMS/GS composite	W/m·K	0.46	0.7 wt% GS (graphene films) at 27 °C	[26]
Thermal conductivity of PDMS/GS foam	W/m·K	0.56	0.7 wt% GS (graphene films) at 27 °C	[26]
Specific heat capacity	kJ/Kg·K	1.46		[23]
Thermal expansion coefficient	K^−1^	20 × 10^−5^	Slight temperature variations	[24]
Melting point	°C	−49.9 to 40		[28]
Glass transition temperature	°C	−125		[29]
Work temperature	°C	−50 to 150		[12]
Mechanical Properties				
Young’s modulus	MPa	0.25–3		[21,29]
Tensile strength	MPa	2.24–6.7		[23,25]
Hardness	Shore A	41–43		[11,30]
Other Physical Properties				
O_2_ diffusion coefficient	cm^2^/s	5.2 × 10^−6^–3.4 × 10^−5^	At 35 °C	[15,31,32]
H_2_O diffusion coefficient	cm^2^/s	1.0 × 10^−5^–2.0 × 10^−4^	At 20 °C and 40% RH	[33,34,35]
Density	g/cm^2^	0.94–0.97	Non-volatile liquid with n * > 7 at room temperature	[12]
Surface tension	dynes/cm^2^	20.1–21.5	Non-volatile liquid with n * > 7 at room temperature	[12]
Vapor pression	mmHg	<10^−5^	At 20 °C Decreases with increasing n *	[6]
Kinematic viscosity	cSt	Low: 0.65 < 50 Medium: 50 < 1000 High: 1000 ≤ 500,000		[6,13]
Other Chemical Properties				
Hydrophobicity	(Contact angle)	113.5 ± 2	Without surface treatment	[9]
Hydrophobicity	(Contact angle)	108.9	O_2_ plasma-treated surface (air-dried)	[9]

* n is the number of repeating units in the PDMS chemical structure. ^†^ polydimethylsiloxane–diphenylsiloxane.

## Data Availability

No new data were created or analyzed in this study.

## Abbreviations

The following abbreviations are used in this manuscript:

PDMS	Polydimethylsiloxane
OFM	Optofluidic Microscope
VOCs	Volatile Organic Compounds
SSL	Solid-State Lighting
FBGs	Fiber Bragg Gratings
